# Differently expressed long noncoding RNAs and mRNAs in TK6 cells exposed to low dose hydroquinone

**DOI:** 10.18632/oncotarget.21481

**Published:** 2017-10-04

**Authors:** Shaoyun Chen, Hairong Liang, Gonghua Hu, Hui Yang, Kairu Zhou, Longmei Xu, Jiaxian Liu, Bei Lai, Li Song, Hao Luo, Jianming Peng, Zhidong Liu, Yongmei Xiao, Wen Chen, Huanwen Tang

**Affiliations:** ^1^ Department of Environmental and Occupational Health, Dongguan Key Laboratory of Environmental Medicine, School of Public Health, Guangdong Medical University, Dongguan, 523808, China; ^2^ Department of Preventive Medicine, Gannan Medical University, Ganzhou, 341000, China; ^3^ Huizhou Prevention and Treatment Centre for Occupational Disease, Huizhou, 516000, China; ^4^ Guangzhou Key Laboratory of Environmental Pollution and Health Risk Assessment, Department of Toxicology, School of Public Health, Sun Yat-sen University, Guangzhou, 510080, China

**Keywords:** long noncoding RNA (lncRNA), hydroquinone, high-throughput sequencing, expression profiles, leukemia

## Abstract

Previous studies have shown that long noncoding RNAs (lncRNAs) were related to human carcinogenesis and might be designated as diagnosis and prognosis biomarkers. Hydroquinone (HQ), as one of the metabolites of benzene, was closely relevant to occupational benzene poisoning and occupational leukemia. Using high-throughput sequencing technology, we investigated differences in lncRNA and mRNA expression profiles between experimental group (HQ 20 μmol/L) and control group (PBS). Compared to control group, a total of 65 lncRNAs and 186 mRNAs were previously identified to be aberrantly expressed more than two fold change in experimental group. To validate the sequencing results, we selected 10 lncRNAs and 10 mRNAs for quantitative real-time PCR (qRT-PCR). Through GO annotation and KEGG pathway analysis, we obtained 3 mainly signaling pathways, including P53 signaling pathway, which plays an important role in tumorigenesis and progression. After that, 25 lncRNAs and 32 mRNAs formed the lncRNA-mRNA co-expression network were implemented to play biological functions of the dysregulated lncRNAs transcripts by regulating gene expression. The lncRNAs target genes prediction provided a new idea for the study of lncRNAs. Finally, we have another important discovery, which is screened out 11 new lncRNAs without annotated. All these results uncovered that lncRNA and mRNA expression profiles in TK6 cells exposed to low dose HQ were different from control group, helping to further study the toxicity mechanisms of HQ and providing a new direction for the therapy of leukemia.

## INTRODUCTION

Benzene is recognized as a human carcinogen, which is a widely used occupational harmful factor in industry [1]. The international agency for research on cancer confirms that benzene is a human cause of leukemia, and hematopoietic system is the target organ of benzene toxicity [2]. Hydroquinone (HQ) is an alternative to benzene *in vitro*. Moreover, a case of acute myeloid leukemia (AML) caused by exposure to HQ for 16 years has been reported recently [3]. Several studies have reported that short-term exposure to HQ induced oxidative DNA damage and apoptosis [4]. Our previous research found that long-term exposure to HQ could result in transforming capability *in vitro* and the tumorigenesis capability *in vivo* [5]. Furthermore, our data have shown that HQ-induced activation of myeloproliferative leukemia virus oncogene was connected with DNA hypomethylation [6]. Thus it can be seen that HQ poses a great threat to human health in the case of unavoidable contact. Making an intensive study on the mechanisms of HQ toxicity is helpful for the early diagnosis and therapy of leukemia. There is an urgent need to improve early detection and identify new targets for therapy.

Long noncoding RNAs (lncRNAs) are defined as newly-identified class of noncoding RNAs (ncRNAs) longer than 200 nucleotides, which were regarded as transcription noise for lacking of open reading frame (ORF) and non-protein coding function [7–9]. Accumulating evidences have indicated that an increased abundance of lncRNAs participating in a variety of biological processes, such as transcription, splicing, translation, chromatin modification, cell growth, cell cycle regulation, apoptosis [10, 11, 8]. Meanwhile, several authors have proposed that lncRNAs can be used as competing endogenous RNAs (ceRNAs) to competitively sponge miRNAs to regulate target genes [12, 13]. With the maturity of the sequencing platform, more and more unknown lncRNAs have entered the academic field of view and attracted the interest of researchers. According to LNCipedia 4.1, the latest version of this long non-coding RNA database contains 146,742 human annotated lncRNAs. Several researchers have revealed that lncRNAs were dysregulated in blood samples of leukemia patients (*in vivo*) and leukemia cells (*in vitro*), which involved in hematopoietic system toxicity [14, 15]. To some extent, lncRNAs as the biomarkers of disease diagnosis and prognosis have become an integral part of the study. Scholars will uncover the mystery of lncRNAs and tap its little-known functions gradually.

With the depth and development of researches, the study of lncRNAs has gradually shifted from human diseases to environmental exogenous chemicals. In this study, we performed profiles of differentially expressed lncRNAs and mRNAs in 2 pairs of low dose experimental group (HQ 20 μmol/L) and control group (PBS) (repeat 3 times). We also established co-expression networks for differentially expressed lncRNAs and mRNAs, with a view to comprehensively investigating the biological functions of lncRNAs. In addition, we have found several new lncRNAs that have not yet been annotated in major databases, suggesting that there is a possibility of a major breakthrough.

## RESULTS

### Total RNA purity, concentration and quality identification of samples

**Figure 1:**
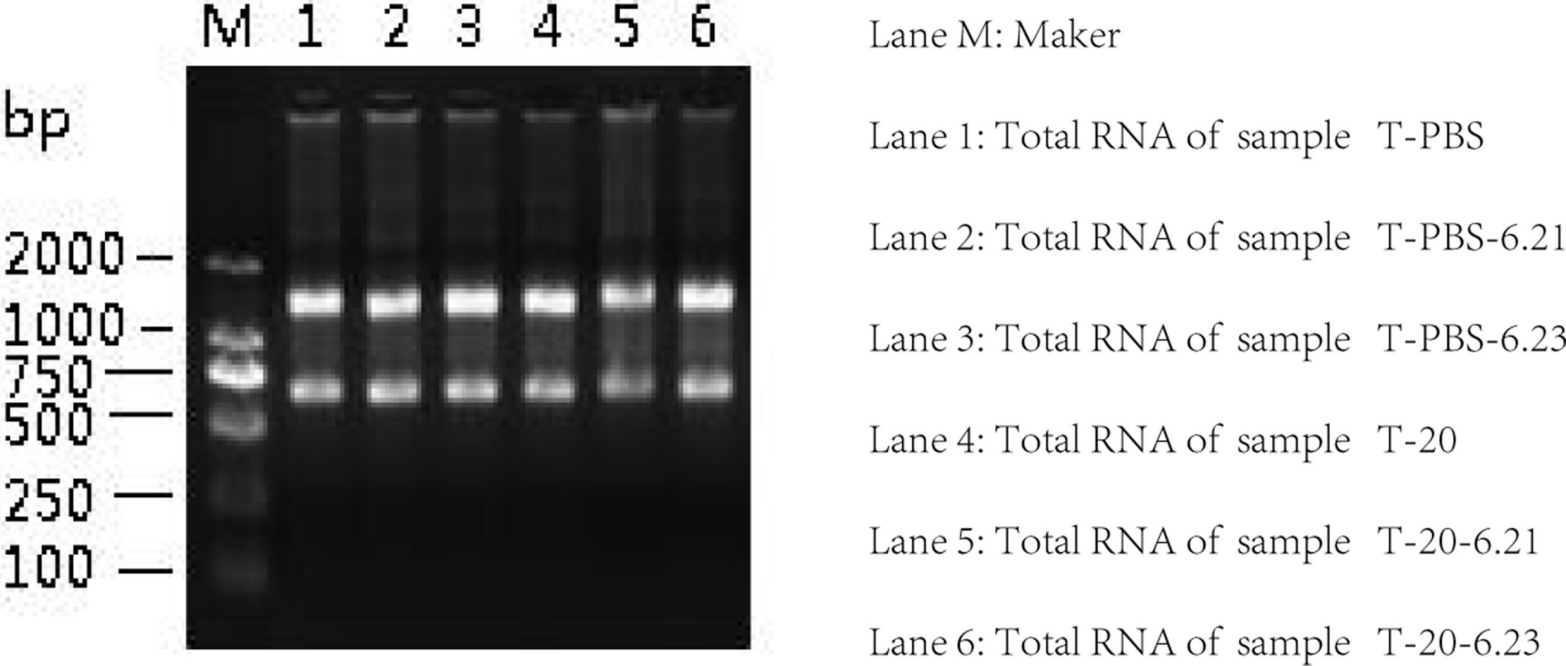
Total RNA agarose gel electrophoresis

PBS and HQ (20 μmol/L) treated TK6 cells in a total of two sets (3 replicates per group) in our study. Quality inspection results have shown that the total RNA purity (A260/A280) of all samples was about 2.0 (Table 1), indicating that there was no protein residue. After the denatured RNA electrophoresis of the total RNA, the bands were clearly observed on the gel imaging system, indicating that the quality of the total RNA was good, and there was no obvious degradation (Figure 1). To sum up, all total RNA were suitable for further analysis.

**Table 1:** All samples of total RNA quality test results

Sample ID	Sample name	K5500			AGE	Agilent 2200		Detection conclusion
		OD260/280	OD260/230	Total amount (ug)		RIN	28S/18S	
1	T-PBS	1.87	2.00	15.99	pass	10.0	3.2	A
2	T-PBS-6.21	1.85	1.91	21.04	pass	10.0	3.0	A
3	T-PBS-6.23	1.90	2.04	28.88	pass	10.0	3.4	A
4	T-20	1.79	2.05	16.38	pass	10.0	3.4	A
5	T-20-6.21	1.90	1.64	22.50	pass	10.0	2.3	A
6	T-20-6.23	1.84	1.98	23.53	pass	10.0	2.8	A

T-PBS, T-PBS-6.21, T-PBS-6.23: PBS solvent control group (3 replicates).

T-20, T-20-6.21, T-20-6.23: 20 μmol/LHQ experimental group (3 repetitions).

A: Indicating that the sample qualified to meet the requirements of the construction of two libraries.

### Differentially expressed lncRNAs and mRNAs in TK6 cells exposed to low dose HQ

**Figure 2:**
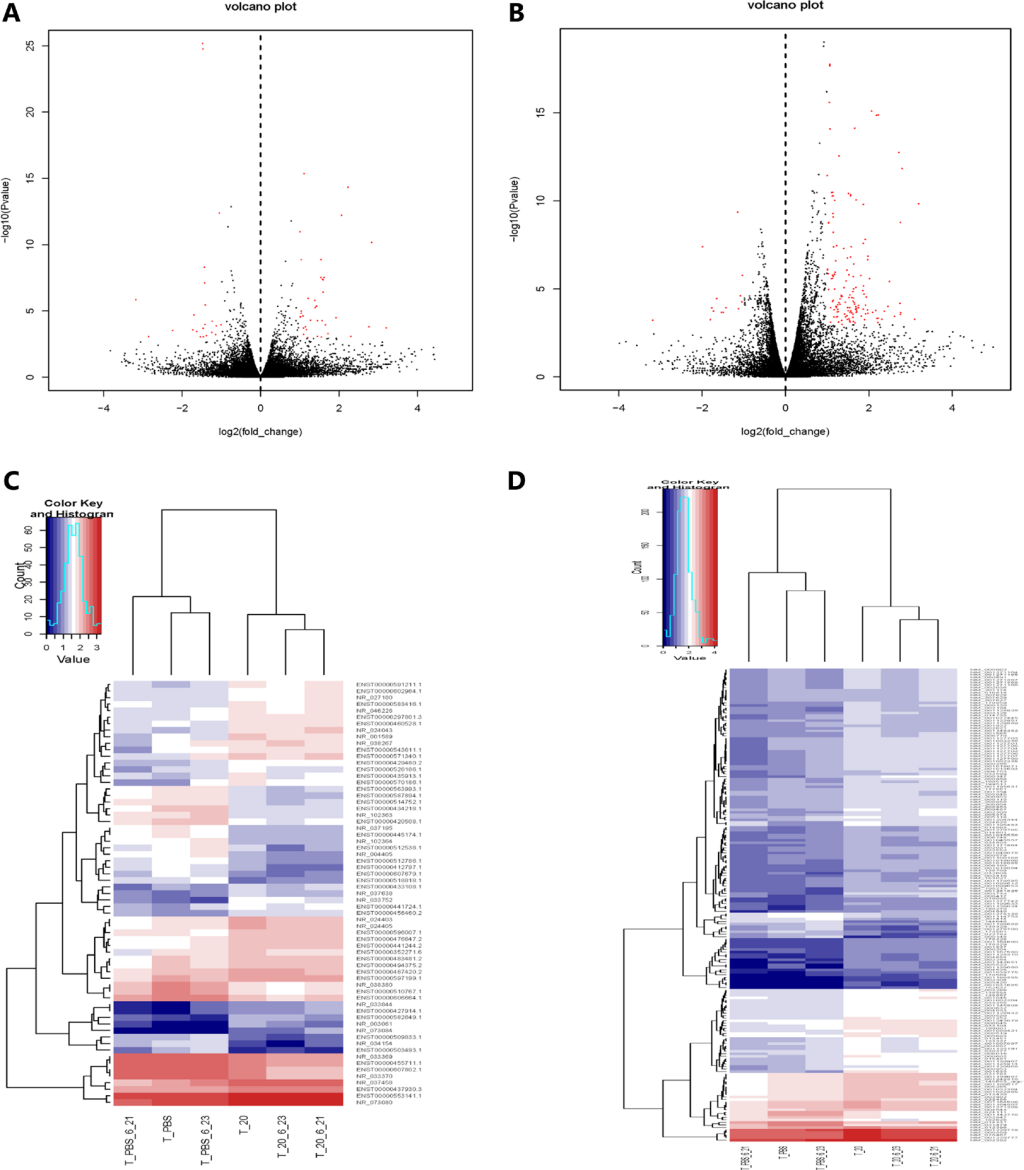
Gene expression profile differences between HQ group compared to PBS group Volcano plots of the differentially expressed lncRNAs (**A**) and mRNAs (**B**). Dark dots represent lncRNAs/mRNAs not significantly differentially expressed (fold change < 2, *P* > 0.05) and red dots represent lncRNAs/mRNAs significantly differentially expressed (fold change ≥ 2, *P* < 0.05). Left and right indicate low and high expression, respectively. Hierarchical clustering indicates lncRNAs (**C**) and mRNAs (**D**) profiles. Red column indicates up regulation and blue column indicates down regulation, red through blue color indicates high to low expression level.

Integration of RNA-seq data is available that 65 differentially expressed lncRNAs from 30632 lncRNAs were statistically significant (FC ≥ 2.0, *P* < 0.05). Among them, 40 lncRNAs were high expression (upregulated) and 25 lncRNAs were low expression (downregulated) (Figure 2A, 2C). We screened 10 lncRNAs with large changes and statistically significant in expression for subsequent studies. Upregulated lncRNAs included LILRP2, LOC344887, LGALS9, HBBP1, ENSG00000270164.1 (LINC01480), while downregulated lncRNAs included C11orf92, LAMB2P1, LOC63930, SLC7A11-AS1, ENSG00000272259.1 (Table 2). Using the same standard as lncRNAs, 186 mRNAs were differentially expressed from 34791 mRNAs (FC ≥ 2.0, *P* < 0.05), including 170 upregulated mRNAs and 16 downregulated mRNAs (Figure 2B, 2D). 5 up and down regulated mRNAs are listed below, respectively (Table 3).

**Table 2:** The five statistically significantly up- and down-regulated lncRNAs

ID	LncRNA	control group (PBS)	experimental group (HQ 20 μmol/L)	Log2FoldChange	*p*-value	*q-*value	Regulation
NR_003061	LILRP2	1.05948	9.76022	3.20356	1.90 × 10^–4^	1.37 × 10^–2^	Up
NR_033752	LOC344887	5.40882	38.56363	2.83385	6.84 × 10^–11^	7.79 × 10^–8^	Up
NR_024043	LGALS9	19.82989	92.79888	2.06227	4.55 × 10^–15^	1.52 × 10^–11^	Up
NR_001589	HBBP1	20.55639	85.85218	2.06227	6.03 × 10^–13^	1.21 × 10^–9^	Up
ENST00000597199.1	ENSG00000270164.1 (LINC01480)	108.61434	223.18248	1.03901	1.52 × 10^–5^	2.30 × 10^–3^	Up
NR_034154	C11orf92	19.38408	2.14231	−3.17763	1.42 × 10^–6^	3.75 × 10^–4^	Down
NR_004405	LAMB2P1	36.53608	11.26306	−1.69772	2.04 × 10^–5^	2.97 × 10^–3^	Down
NR_033370	LOC63930	518.15870	187.23227	−1.46856	1.72 × 10^–25^	4.32 × 10^–21^	Down
NR_038380	SLC7A11-AS1	171.43246	63.52541	−1.43223	5.18 × 10^–9^	3.37 × 10^–6^	Down
ENST00000607802.1	ENSG00000272259.1	523.26882	188.03938	?−1.47652	6.83 × 10^–26^	3.42 × 10^–21^	Down

**Table 3:** The five statistically significantly up- and down-regulated mRNAs

ID	mRNA	control group (PBS)	experimental group (HQ 20 μmol/L)	Log2FoldChange	*p*-value	q-value	Regulation
NM_001633	AMBP	25.70499	235.22885	3.19394	1.49 × 10^–10^	1.49 × 10^–7^	Up
NM_178564	NRBP2	1.11449	9.53745	3.09722	5.40 × 10^–4^	3.07 × 10^–2^	Up
NM_198213	OASL	7.02611	48.85791	2.79779	1.49 × 10^–12^	2.87 × 10^–9^	Up
NM_001142651	NEURL1B	1.95092	13.25272	2.76406	2.27 × 10^–4^	1.58 × 10^–2^	Up
NM_001039775	AIM1L	1.82081	12.31208	2.75742	2.90 × 10^–4^	1.89 × 10^–2^	Up
NM_000148	FUT1	38.96812	4.29962	-3.18001	6.11 × 10^–4^	3.38 × 10^–2^	Down
NM_144646	IGJ	63.50211	15.99137	-1.98951	4.06 × 10^-8^	1.93 × 10^–5^	Down
NM_022783	DEPTOR	23.45508	6.72155	-1.80304	5.68 × 10^–4^	3.19 × 10^–2^	Down
NM_001142776	CHAC1	503.88556	147.97588	-1.76773	1.24 × 10^–4^	1.00 × 10^–2^	Down
NM_031479	INHBE	935.28009	297.98525	-1.65016	3.61 × 10^–5^	4.29 × 10^–3^	Down

### Sequencing results validation

**Figure 3:**
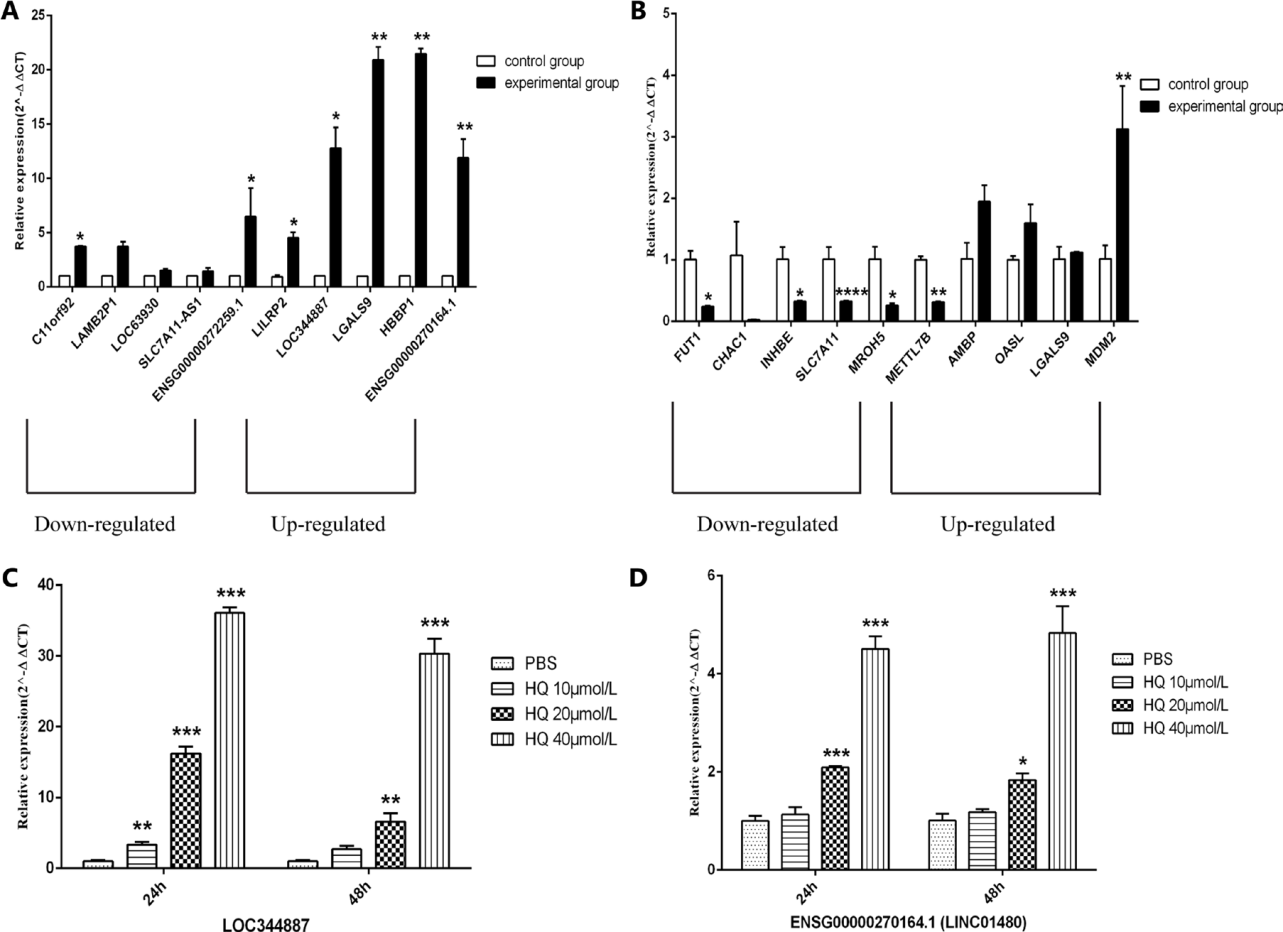
qRT-PCR validation of the sequencing results qRT-PCR validation of 10 differentially expressed lncRNAs (**A**) and mRNAs (**B**). Control group represents PBS, experimental group represents HQ 20μmol/L. (A) Compared to control group, c11orf92, ENSG00000272259.1, LILRP2, LOC344887, LGALS9, HBBP1, ENSG00000270164.1 were significantly differentially expressed. Among them, LILRP2, LOC344887, LGALS9, HBBP1, ENSG00000270164.1 expression changes consistent with the sequencing results. (B) Compared to control group, FUT1, INHBE, SLC7A11, MROH5, METTL7B, MDM2 were significantly differentially expressed. Among them, FUT1, INHBE, SLC7A11, MROH5, MDM2 expression changes consistent with the sequencing results. The expressions of LOC344887 (**C**) and ENSG00000270164.1 (LINC01480) (**D**) were validated by qRT-PCR at different concentrations of HQ (24h and 48h). *P < 0.05, **P < 0.01, ***P < 0.001, ****P < 0.0001.

On the one hand, in order to validate the sequencing results, on the other hand, for the further in-depth research, we selected 10 differentially expressed lncRNAs and mRNAs of interest for qRT-PCR, respectively. Our results have shown that the changes were statistically different for only 7 of the 10 lncRNAs transcripts and 6 of the 10 mRNAs. Among 7 lncRNAs transcripts (c11orf92, ENSG00000272259.1, LILRP2, LOC344887, LGALS9, HBBP1 and ENSG00000270164.1), 5 lncRNAs transcripts (LILRP2, LOC344887, LGALS9, HBBP1 and ENSG00000270164.1) were same trend as the sequencing results. The sequencing data showed that the expression of C11orf92 and ENSG00000272259.1 were upregulated, while qRT-PCR was verified to be down-regulated. LILRP2, LOC344887, LGALS9, HBBP1, ENSG00000270164.1 (LINC01480) were upregulated as shown in the Figure 3A. And among 6 mRNAs (FUT1, INHBE, SLC7A11, MROH5, METTL7B and MDM2), the expression trends of 5 mRNAs (FUT1, INHBE, SLC7A11, MROH5 and MDM2) were consistent with the sequencing results (Figure 3B). In addition, the expression levels of LOC344887 and ENSG00000270164.1 (LINC01480) were validated by qRT-PCR at different concentrations of HQ (PBS, 10 μmol/L, 20 μmol/L, and 40 μmol/L). The results showed that the expression levels of LOC344887 and ENSG00000270164.1 (LINC01480) increased with the increase of HQ concentration after 24h or 48h in TK6 cells exposed to different concentrations HQ (Figure 3C, 3D).

### GO and KEGG pathway analysis

**Figure 4:**
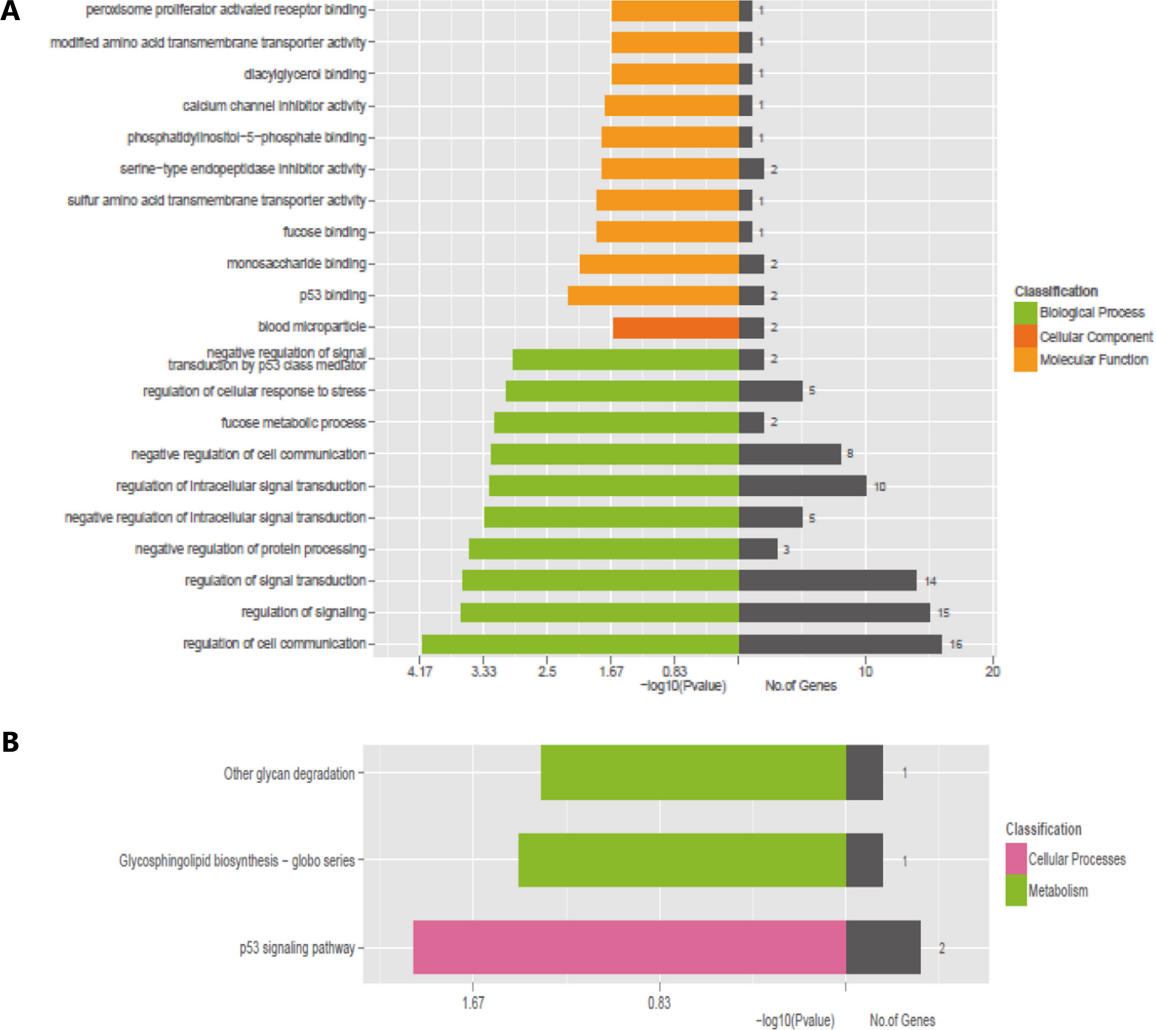
GO and KEGG pathway analysis for differentially expressed mRNAs (**A**) GO analysis for differentially expressed mRNAs. (**B**) KEGG pathway analysis for differentially expressed mRNAs.

In general, lncRNAs played biological functions by co-expressed mRNAs because they have no ability to encode proteins. Thence, we used two enrichment analyses, GO and KEGG pathway analysis to predict the potential roles of dysregulated mRNAs. GO annotation results revealed 3 structured networks: biological processes (1065 GO Terms), cellular components (115 GO Terms) and molecular function (155 GO Terms). In 186 differentially expressed mRNAs, we selected 32 mRNAs for GO annotation and KEGG pathway analysis. Among 32 differentially expressed mRNAs, 16 mRNAs (GPR55, ARHGEF6, CDKN1A, NRBP2, LGALS9, GNG7, CHAC1, DEPTOR, MYOF, MDM2, AMBP, EDA2R, SERPINE1, PHLDA3, UNC13A and ZNF385A) participate in regulation of cell communication (biological process), 15 mRNAs (GPR55, ARHGEF6, CDKN1A, LGALS9, GNG7, CHAC1, DEPTOR, MYOF, MDM2, AMBP, EDA2R, SERPINE1, PHLDA3, UNC13A and ZNF385A) participate in regulation of signaling (biological process). 2 mRNAs (IGJ, AMBP) are involved in blood microparticle (cellular component), 4 mRNAs (STON2, ZNF385A, MDM2, OASL) are involved in nucleolus (cellular component). 2 mRNAs (MDM2, ZNF385A) are related to p53 binding (molecular function), 2 mRNAs (FUCA1, LGALS9) are related to monosaccharide binding (molecular function) (Figure 4A). KEGG database indicated the differentially expressed mRNAs mainly significantly participate in the following pathways: p53 signaling pathway (2 mRNAs: SERPINE1, MDM2), glycosphingolipid biosynthesis-globo series and Glycosphingolipid biosynthesis - lacto and neolacto series (1 mRNA: FUT1) and other glycan degradation (1 mRNA: FUCA1) and so on (Figure 4B).

### LncRNA-mRNA co-expression network analysis and lncRNAs target genes prediction

**Figure 5:**
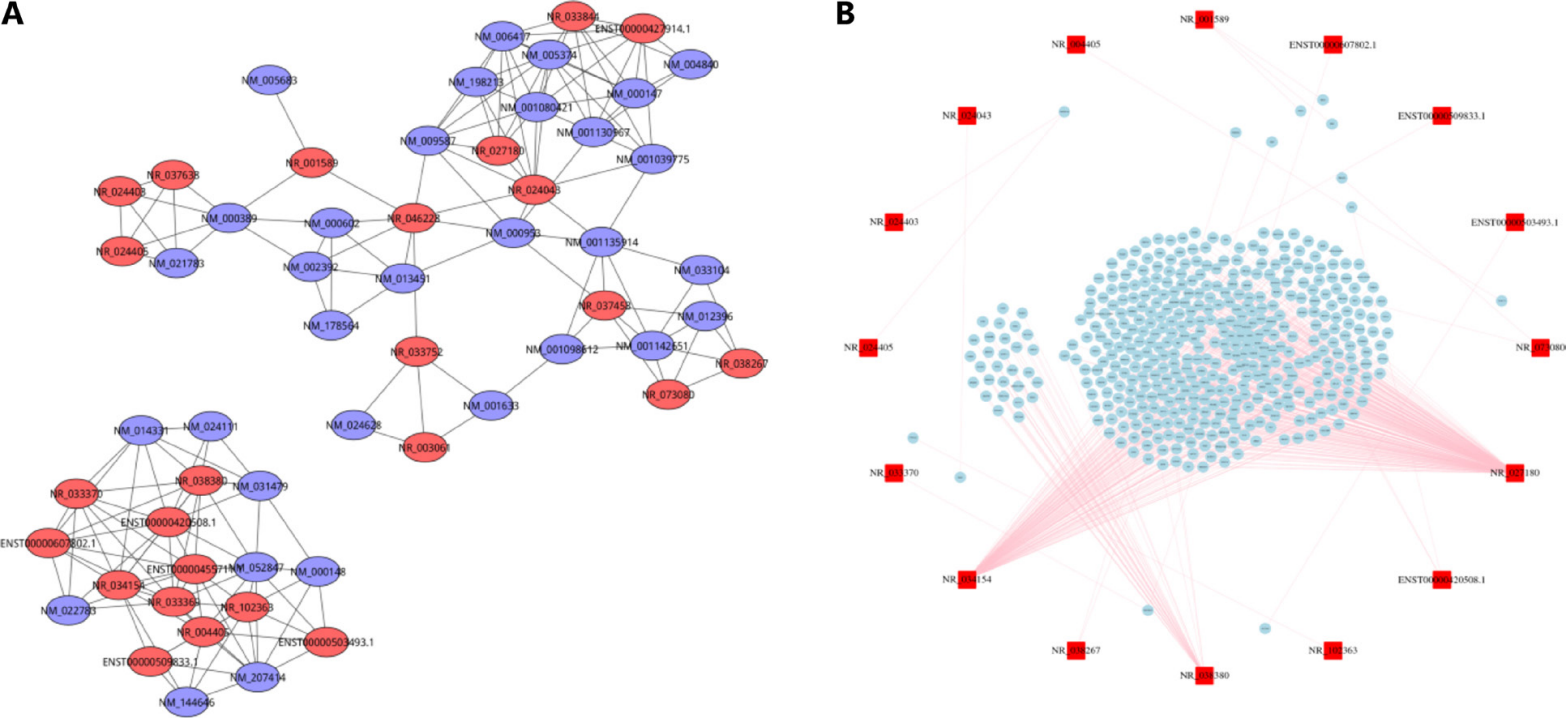
LncRNA-mRNA co-expression network and lncRNA target prediction (**A**) LncRNA-mRNA co-expression network. The red circles represent lncRNAs and the purple rings represent mRNAs. (**B**) LncRNA target prediction. The red squares represent lncRNAs and the blue squares represent mRNAs. The pink lines represent the targeting relationship of lncRNA-mRNA.

We screened 25 lncRNAs and 32 mRNAs to construct the lncRNA-mRNA co-expression network. We could see that a single lncRNA could be co-expressed with tens of the differentially expressed mRNA, we set a strict standard to build the most reliable lncRNA-mRNA co-expression pairs through the Pearson correlation value and *p* value (Figure 5A). In order to gain a better understanding of the biological functions of lncRNAs, we predicted the target genes of the dysregulated lncRNAs by target prediction softs. We selected 27 lncRNAs for target genes prediction, and the results showed that 18 lncRNAs had target genes and 9 had no target genes. Among 18 lncRNAs, 13 lncRNAs had *cis* target genes, and 5 lncRNAs had *trans* target genes (Figure 5B, Table 4).

**Table 4:** The statistical results of lncRNAs target genes

LncRNA	Cis_Num	Trans_Num
NR_046228	0	0
NR_073080	0	2
NR_037195	0	0
NR_004405	1	0
NR_033752	0	0
NR_033844	0	0
NR_038380	1	35
NR_027180	0	280
NR_024405	1	0
NR_024403	1	0
NR_102363	1	0
NR_037638	0	0
NR_001589	3	0
NR_034154	2	137
NR_024043	1	0
NR_038267	2	0
NR_003061	0	0
NR_033369	0	0
NR_033370	1	0
NR_037458	0	0
ENST00000427914.1	0	0
ENST00000503493.1	1	0
ENST00000509833.1	1	0
ENST00000597199.1	0	0
ENST00000607802.1	1	0
ENST00000455711.1	0	0
ENST00000420508.1	0	3

### New lncRNAs prediction

**Figure 6:**
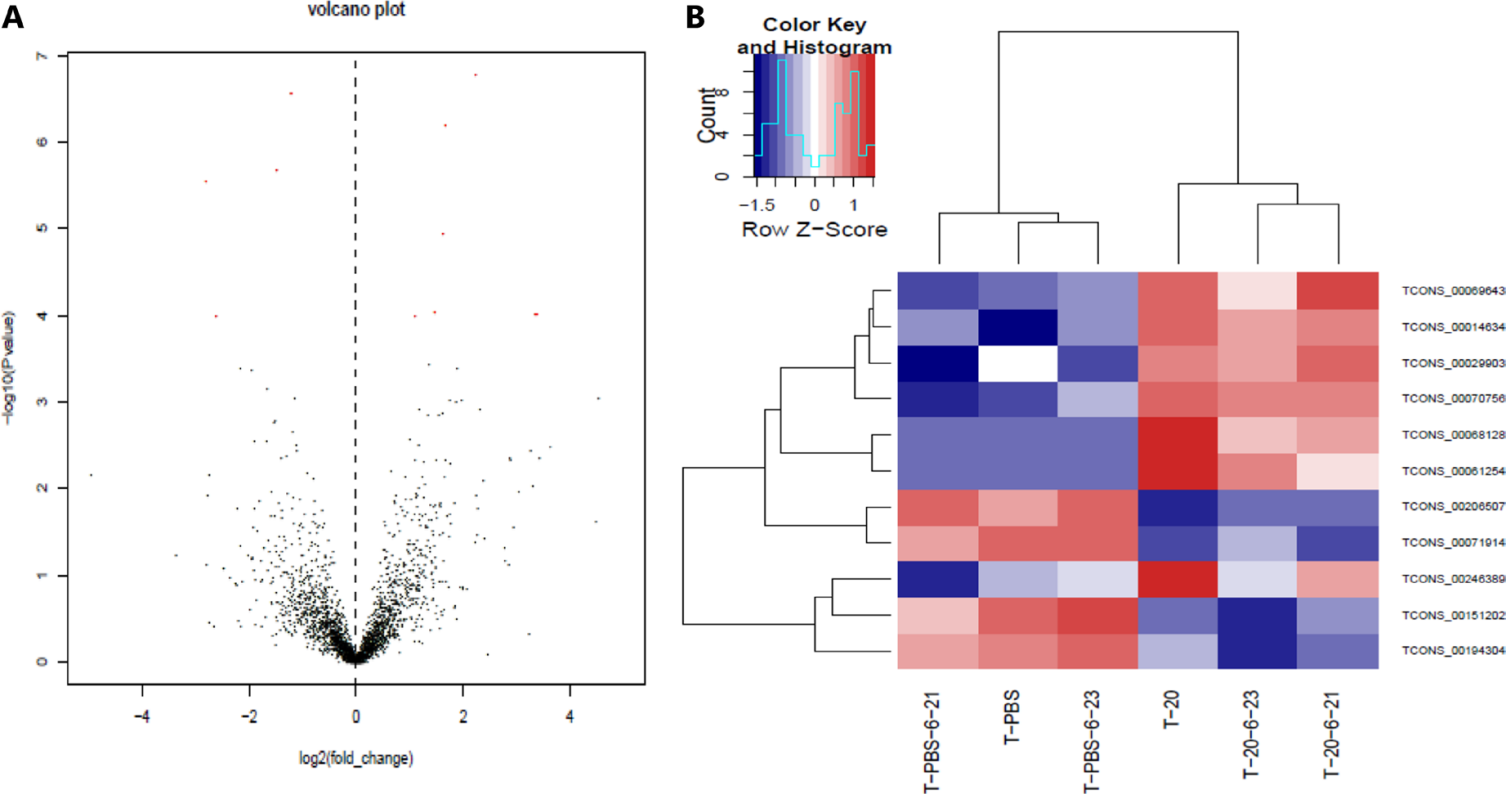
New lncRNAs expression profile differences between HQ group compared to PBS group (**A**) Volcano plots of the differentially expressed new lncRNAs. The dark dots represent new lncRNAs not significantly differentially expressed (fold change < 2, *P* > 0.05) and red dots represent new lncRNAs significantly differentially expressed (fold change ≥ 2, *P* < 0.05). Left and right indicate up and down regulation. (**B**) Hierarchical clustering indicates new lncRNAs. Red through blue color indicates high to low expression level.

Besides the known lncRNAs, our sequencing results also included 11 new lncRNAs as shown in the following table (Table 5). Same as the known lncRNAs mentioned above, we also constructed the volcanic plot (Figure 6A) and the heat map (Figure 6B) of the differentially expressed new lncRNAs. From the table and the figures, we could see that 7 and 4 new lncRNAs are upregulated and downregulated, respectively.

**Table 5:** The statistically significantly up- and down-regulated new lncRNAs

ID	Postion	control group (PBS)	experimental group (HQ 20 μmol/L)	Log_2_FoldChange	*p-*value	q-value	Regulation
TCONS_00061254	chr13:95144393-95146015	1.79690	18.55675	3.36836	9.83 × 10^-5^	0.02141	Up
TCONS_00068128	chr14:52653466-52654526	1.79690	18.20023	3.34038	9.47 × 10-5	0.02141	Up
TCONS_00070756	chr14:52701056-52702850	9.53822	44.80731	2.23194	1.67 × 10-7	0.00033	Up
TCONS_00069643	chr14:52707081-52709855	19.53592	62.28355	1.67272	6.46 × 10-7	0.00050	Up
TCONS_00014634	chr1:238415975-238418276	16.60542	50.97449	1.61812	1.15 × 10-5	0.00446	Up
TCONS_00029903	chr10:85308174-85309741	16.81493	46.36284	1.46323	9.19 × 10-5	0.02141	Up
TCONS_00246389	chrUn_gl000214:53120-53874	37.96233	80.98600	1.09310	1.01 × 10-4	0.02141	Up
TCONS_00194304	chr5:173097914-173101397	203.29403	87.20235	-1.22113	2.78 × 10-7	0.00033	Down
TCONS_00151202	chr22:35848545-35849939	72.85973	25.95650	-1.48903	2.07 × 10-6	0.00121	Down
TCONS_00071914	chr14:69096123-69097892	21.60610	3.51209	-2.62104	9.99 × 10-5	0.02141	Down
TCONS_00206507	chr6:144391624-144393136	26.45023	3.78821	-2.80369	2.91 × 10^-6^	0.00136	Down

## DISCUSSION

Up to this date, many studies have shown that lncRNAs played crucial biological roles in human diseases, such as cancer [16, 17], cardiovascular diseases [18] and neuropathic pain [19]. However, there are few studies about lncRNAs involving in exogenous compounds in the environment. Therefore, the study on functions and mechanisms of lncRNAs in this area is limited. In contrast to microarray, the next-generation sequencing techniques were allowed for faster and accurate to gain the differentially expressed profiles of genes. Because it doesn't need to synthesis probes, which sequenced directly [20]. In addition, to our knowledge, no obvious research has focused on the RNA-Seq expression profile of lncRNAs in HQ treatment. In view of the above considerations, we used RNA-Seq techniques to profile lncRNAs in experimental group (HQ 20 μmol/L) and control group (PBS).

Our previous studies about HQ toxicity mechanisms results mainly involved in autophagy [21], cell cycle and apoptosis [22] and malignant transformation [23]. For a more comprehensive understand the toxicity mechanisms of HQ, we treated TK6 cells from the spleen of hereditary red blood cells anemia patients with exogenous compound (20 μmol/L HQ) as the experimental group in this study. TK6 lymphoblastoid cells line express wild type p53 and being a commonly used model for HQ and/or leukemia researches. Based on the actual level of human contact with HQ and preliminary studies, TK6 cells were treated with HQ 20μmol/L. Our results are the first RNA-Seq analysis indicating 65 lncRNAs and 186 mRNAs dysregulated in HQ treatment with fold changes of two or more. Compared with the control group (PBS), LILRP2, LOC344887, LGALS9, HBBP1 and ENSG00000270164.1 (LINC01480) are upregulated and C11orf92, LAMB2P1, LOC63930, SLC7A11-AS1, ENSG00000272259.1 are low expression in RNA-Seq data. We verified that the up-regulation of lncRNAs trends was consistent with sequencing results, while the down-regulation of lncRNAs trends was different from sequencing results. The expression trends of C11orf92 and ENSG00000272259.1 were contrary to sequencing results and the changes were statistically significant. Furthermore, FUT1, INHBE, SLC7A11, MROH5, METTL7B and MDM2 were significantly differentially expressed, while only METTL7B were contrary to sequencing data. In Figure 3C, 3D, the results showed that the expressions of LOC344887 and ENSG00000270164.1 (LINC01480) increased with the increase of HQ concentration after 24h or 48h in HQ treated TK6 cells at different concentrations. Wu et al. [24] has demonstrated that LOC344887 was amplified in gallbladder cancer and promoted epithelial mesenchymal transition (EMT) . Silencing of LOC344887 via CRISPR/Cas9 genome-editing protected against sulforaphane (SFN) -mediated inhibition of cancer cell growth, colony formation, and migration [25]. There is no literature reported ENSG00000270164.1 (LINC01480) function. Therefore, we can make a hypothesis that LOC344887 and ENSG00000270164.1 (LINC01480) may be involved in HQ induced TK6 cytotoxicity mechanisms. The specific mechanisms or signaling pathways need further exploration, and are also the focus of our next research. With the advantages of high signal-to-noise ratio, high resolution and wide application range, the new generation sequencing technology also has some errors from sample contamination and sequencing errors [26].

It is worth mentioning that lncRNAs can serve as signal, decoy, guide or scaffold molecules to participate in pre-transcriptional regulation, transcriptional regulation and post-translational regulation [27, 26, 11]. LncRNAs usually regulate target genes to perform their biological function because of lacking of proteins encode ability. Thus, a comprehensive understanding of the potential function for differentially expressed mRNAs is essential. Through GO annotation and KEGG pathway analysis, we could identify potential functions of the dysregulated mRNAs. GO annotation [28] revealed that the vast majority of differentially expressed mRNAs involve in multiple biological processes. IGJ and AMBP involved in cellular components. Simultaneously, partial mRNAs were involved in molecular functions, such as p53 binding, monosaccharide binding and fucose binding. KEGG database [29] revealed that the dysregulated mRNAs mainly involve in HQ toxicity mechanism through p53 signaling pathway and Glycosphingolipid biosynthesis - globo series pathway. Among them, chronic myeloid leukemia may be associated with MDM2 abnormalities. As the target gene of p53, MDM2 played an important role in the ubiquitin-proteasome pathway [30, 31]. Surprisingly, the combination of lincRNa-p21 and MDM2 promoted p53 ubiquitination, that is to say lincRNa-p21 positively regulated MDM-p53 interaction [32]. Studying the potential function of the differentially expressed mRNAs, can help to gain insight into their involvement in signaling pathways and provide convenience for researchers in the study of lncRNAs, simultaneously.

With the maturity of sequencing technology, more and more lncRNAs have been discovered and annotated, but their functional researches are still limited. Due to considerable difficulties in making a thorough inquiry on the lncRNAs functions directly, it is particularly important to predict lncRNAs target genes and construct lncRNA-mRNA co-expression network according to a co-expression-based method [33, 18]. LncRNAs regulated the expression of co-expressed mRNAs mainly through *cis* and *trans*. For instance, lncRNA-HBBP1 (NR_001589) regulated the expression of HBG1, HBG2, HBD by *cis* regulation and lncRNA-PHLDA3 (NR_073080) *trans* regulated the expression of CYP20A1 and ZNF470. The HBG1 and HBG2 are normally expressed in the fetal liver, spleen and bone marrow. Compared with wild type promoter, the activity of HBG1 gene promoter variant decreased significantly in K562 cells under conditions of erythropoietic stress [34]. This finding is helpful to analyze the pathogenesis of β-Thalassemia and provide a reference for its treatment. Because HBG1 and HBG2 are involved in the pathogenesis of hematologic diseases, it is not difficult to imagine that lncRNA- HBBP1 may also be involved in hematologic diseases and even leukemia. In view of the long-term exposure to HQ could lead to blood system diseases, then this coincide with our initial hypothesis. Another advantage of RNA-Seq technology is the ability to filter out new lncRNAs without annotated. We have identified 11 new lncRNAs, which will provide a new breakthrough for the studies of HQ toxicity mechanisms and leukemia pathogenesis.

More and more literatures have proved that lncRNAs were closely associated with the pathogenesis of leukemia. We have made a review about the lncRNAs as the novel diagnostic biomarkers of leukemia. For instance, MEG3, RUNXOR, NEAT1, LLEST, IRAIN and UCA1 were related to AML; ANRIL, LUNAR1 and T-ALL-R-LncR1 were involved in ALL; LncRNA-BGL3, H19 and MEG3 participated in CML; lincRNA-p21, DLEU1/DLEU2 and TRERNA1 were involved in CLL [35]. However, the functions of more lncRNAs we sequenced by RNA-Seq has not been studied. Next, we plan to take the blood samples from patients with occupational leukemia for sequencing in order to find the lncRNAs consistent with this paper.

## MATERIALS AND METHODS

### Cell culture and HQ treatment

TK6 cells are kindly provided by Professor Lishi Zhang of West China Medical College of Sichuan University, and were cultured at 37°C in an atmosphere containing 5% CO_2_ in RPMI1640 medium (Gibco Invitrogen, UK) plus 10% horse serum (HS). HQ (purity > 99%, Sigma, St. Louis, MO) was dissolved in phosphate-buffered saline (PBS) and immediately added to TK6 cells (20 μmol/L), the cells were cultured for 48h.

### RNA isolation and qRT-PCR

Total RNA of cells was isolated using Trizol reagent (Invitrogen, Carlsbad, CA, USA) following the manufacturer's instructions. The RNA concentrations were quantified by NANODROP 2000c (Thermo Scientific, USA), and the quality of RNA was assessed by 2% agarose gel electrophoresis. Extracted total RNA was used to synthesize cDNA using Transcriptor First Strand cDNA Synthesis Kit (Roche, Mannheim, Germany). qRT-PCR reactions were performed in triplicate in a 96-well plate containing 1μl of synthesized cDNA (diluted 6 times), FastStart Universal SYBR Green Master (Roche, Mannheim, Germany) on PikoReal (Thermo Scientific, USA) in a total volume of 10μL. The expression levels of lncRNAs were normalized to GAPDH and calculated using the 2^−ΔΔCt^ method. All of primers were designed and synthesized by Generay Biotechnology (Generay Biotechnology, Shanghai) (Table 6).

**Table 6:** PCR primers

Gene	Primer sequence (5′–3′)
C11orf92	F:GCCTCTGCTGTTTATTCTCT
	R:CTCTGTGTGTTTCCCTCTGT
LAMB2P1	F:GAGGTGTGTGTGATGTGTGT
	R:GTGTTGGGATTAGGGGTT
LOC63930	F:ATGGGAGCGGGTAGATAA
	R:GAGACAGGAAGAGAGAGCAG
SLC7A11-AS1	F:CAAAATGCCTGCTAAAGTG
	R:CGCTGTGAAATCTGTCTGT
ENSG00000272259.1	F:ATCTCTCCTTCTGGCTGTG
	R:TCCTGTTGTTCCTGCTGT
LILRP2	F:AGAGACGAGGGACAGGAG
	R:GGATTTGGAACACAGAGGT
LOC344887	F:CTATGAAATGAAGCCAGACC
	R:GTGCCGTAAAGAGGAAAAG
LGALS9	F:CCATCCAAGTCCATCCTC
	R:TAGTATTCAAACAGGTGCTGAC
HBBP1	F:GAGAAGGCTGGAGGTGAG
	R:GTCATCCGTGAGCATAACA
ENSG00000270164.1 (LINC01480)	F:AGGAGAAAGGCATAGCAGA
	R:CAGGAAAGGGAGAGAAGTG
GAPDH	F:TCTGACTTCAACAGCGACACC
	R:CTGTTGCTGTAGCCAAATTCGT
FUT1	F: GGCGTGCGGCTGTCCACTTCT
	R: TGGCTCTGGCCCAGCTCAACG
CHAC1	F: GCCAGACGCAGCAAGTATTCA
	R: GAGGTCACCTTCTATCCCCAAGA
INHBE	F: ACACCAGACTTCTCACCCCTC
	R: CTCCCTGCTCACTTTTCACCT
SLC7A11	F: CACATTTGTCAGCACATAGCC
	R: ACTGAAGAAGTAGAAAACCCTG
MROH5	F: GCTGGGCTCAGGGTCACTTCT
	R: ACAGCCACTCCTGCCACATCA
METTL7B	F: GATTTGGGTCTAGGCAGGTGA
	R: CAGATAAAGGGGCTTACAGGA
AMBP	F: GACACTCCTTTTCTGTGACGA
	R: ATGACCAGCAGGTATTTCTAT
OASL	F: CAGGGCTCTGTAGGCAGGCAC
	R: GGAGGCAGCCAAGCATCACAA
LGALS9	F: CAGACTTCGCTCCTCAGACCC
	R: TGCCTTTCATCACCACCATTC
MDM2	F: AAAGGGAAGAAACCCAAGACAAAGA
	R: GCCATGTAAAGCAGGCCATAAGA

### High-throughput sequencing and data set

Total RNA was isolated from cells/tissues using the Trizol (invitrogen) according to the manufacturer's protocol. RNA purity was assessed using the Qubit^®^. Each RNA sample had an A260:A280 ratio above 1.8 and A260:A230 ratio above 2.0. RNA integrity was evaluated using the Agilent 2200 TapeStation (Agilent Technologies, USA) and each sample had the RINe above 7.0. Briefly, rRNAs were removed from Total RNA using Epicentre Ribo-Zero rRNA Removal Kit (illumina, USA) and fragmented to approximately 200bp. Subsequently, the purified RNAs were subjected to first strand and second strand cDNA synthesis following by adaptor ligation and enrichment with a low-cycle according to instructions of TruSeq® RNA LT/HT Sample Prep Kit (Illumina, USA). The purified library products were evaluated using the Agilent 2200 TapeStation and Qubit^®^2.0 (Life Technologies, USA) and then diluted to 10 pM for cluster generation *in situ* on the HiSeq3000 pair-end flow cell followed by sequencing (2 × 150 bp) on HiSeq 3000. Finally, the obtained data were analyzed by bioinformatics. All RNA-Seq data was aligned to hg19 using TopHa v1.4 [36] with default parameters. We used Cuffdiff v1.3 [37] to analyze differentially expressed genes and obtained custom annotations composed of RefSeq entries plus HQ-induced TK6 lncRNAs as described below. The difference was considered statistically significant when *P* < 0.05. To screen the differentially expressed genes, we used threshold values of ≥ 2 (up-regulation) and ≤ -2 (down-regulation) fold change (FC). FC refers to the ratio of FPKM between the experimental group and the control group. FPKM is a general method of gene expression levels, which stands for the fragments Per Kilobase of transcript Per Million mapped reads.

### Hierarchical clustering analysis

Cluster analysis of differential genes was used to determine the clustering patterns of regulation patterns under different experimental conditions. The difference in FPKM for different samples is used for cluster analysis.

### Gene function analysis

The functional annotation of lncRNAs is mainly predicted by their co-expression mRNAs, but there is still no functional annotation of most lncRNAs at present [38]. GO (Gene Ontology) [39] and KEGG (Kyoto Encyclopedia of Genes and Genomes) pathway [40] annotations are useful for analyzing differentially expressed lncRNAs related mRNAs functions. GO analysis is mainly composed of cellular components, biological processes and molecular functions. Pathways about the mRNAs associated with dysregulation of lncRNAs can be obtained by KEGG database besides.

### LncRNA-mRNA co-expression network analysis

The co-expression network was constructed between significantly dysregulated lncRNAs and mRNAs. Considering the influence of random factors, we found that only using Pearson correlation coefficients (PCC) is not strict, so we calculate zscore values and then obtain *P* values. The co-expressed lncRNAs and mRNAs pairs with |COR| > 0.85 and *P* < 0.05 were selected to draw the network. By selecting specific lncRNAs from dysregulated lncRNAs, we mapped the network graphs that interact with their target genes, providing a reference and help for the holistic analysis the function of lncRNAs in the samples.

### LncRNAs target genes prediction

LncRNAs play the biological functions mainly by regulating their target genes because they lacked protein coding ability [41]. The target genes here are also known as transcription factors (TF). We mainly use TFSearch software (http://www.cbrc.jp/research/db/TFSEARCH.html) to predict TF of lncRNAs, and we draw lncRNA-mRNA interactive analysis diagrams through Cytoscape. Moreover, the mechanisms of lncRNAs are similar to miRNAs and can be performed by regulating the corresponding mRNAs. LncRNAs regulate target genes mainly through *cis* and *trans* regulation. The principle of *cis* regulation mechanism: for the differentially expressed lncRNAs, search all the coding genes of their upstream and downstream 10 kb range, whichever is selected with a significant co-expression of the lncRNA. The principle of *trans*-action is mainly based on nucleic acid base pairing.

### New lncRNAs prediction

With the strict definition of lncRNAs, we can screen out new lncRNAs by setting some filtering conditions. The filter conditions are as follows: 1) Filtering database known genes and lncRNAs; 2) FPKM > 2; 3) ORF < 300; 4) Filtering RNAs with a protein domain (Pfam); 5) RNAs with a length greater than 200 nt; 6) Filtering RNAs with a coding potential [42]. An ANNOVAR software (http://www.openbioinformatics.org/annovar/) built-in database is adopted to annotate new lncRNAs.

### Statistical analysis

All experiments were repeated at least 3 times independently, and all values were presented as mean ± standard deviation (SD). The data of two experimental groups were compared using the Student's *t*-test, while One-way ANOVA was analyzed for multi-group comparisons. All data were analyzed and plotted using GraphPad Prism 6 (Graph-Pad Software, La Jolla, CA), and a *P* value of 0.05 or less was considered statistically significant, * means *P* < 0.05.

## CONCLUSIONS

Overall, our study confirm that lncRNAs might participate in HQ toxicity mechanism even play an important role in leukemia. Further studies will be needed to persuasively demonstrate and interpret the more accurate role of one or several specific lncRNAs in TK6 cells exposed to low dose HQ. These findings bring us one step closer to a better understanding of leukemia pathogenesis, and lncRNAs may be the novel biomarkers for the diagnosis and therapy of leukemia.

## Abbreviations

HQ: Hydroquinone
AML: acute myeloid leukemia
GO: Gene Oncology
KEGG: Kyoto Encyclopedia of Genes and Genomes
qRT-PCR: quantitative real-time PCR
HS: horse serum
PBS: phosphate buffered saline
ORF: open reading frame
ceRNAs: competing endogenous RNAs
FPKM: Fragments Per Kilobase of transcript per Million mapped reads
PCC: Pearson correlation coefficients
TF: transcription factors
EMT: epithelial mesenchymal transition
SFN: sulforaphane
